# Optical Constants and Band Gap Evolution with Phase Transition in Sub-20-nm-Thick TiO_2_ Films Prepared by ALD

**DOI:** 10.1186/s11671-017-2011-2

**Published:** 2017-03-31

**Authors:** Yue-Jie Shi, Rong-Jun Zhang, Hua Zheng, Da-Hai Li, Wei Wei, Xin Chen, Yan Sun, Yan-Feng Wei, Hong-Liang Lu, Ning Dai, Liang-Yao Chen

**Affiliations:** 1 Key Laboratory of Micro and Nano Photonic Structures, Ministry of Education, Department of Optical Science and Engineering, Fudan University, Shanghai, 200433 China; 2 National Laboratory for Infrared Physics, Shanghai Institute of Technical Physics, Chinese Academy of Sciences, Shanghai, 200083 China; 3 State Key Laboratory of ASIC & System, Fudan University, Shanghai, 200433 China

**Keywords:** TiO_2_ ultrathin film, Atomic layer deposition, Spectroscopic ellipsometry, Rapid thermal annealing, Phase transition

## Abstract

Titanium dioxide (TiO_2_) ultrathin films with different thicknesses below 20 nm were grown by atomic layer deposition (ALD) on silicon substrates at 300 °C. Spectroscopic ellipsometry (SE) measurements were operated to investigate the effect of thickness on the optical properties of ultrathin films in the spectra range from 200 to 1000 nm with Forouhi–Bloomer (F-B) dispersion relation. It has been found that the refractive index and extinction coefficient of the investigated TiO_2_ ultrathin film increase while the band gap of TiO_2_ ultrathin film decreases monotonically with an increase in film thickness. Furthermore, with the purpose of studying the temperature dependence of optical properties of TiO_2_ ultrathin film, the samples were annealed at temperature from 400 to 900 °C in N_2_ atmosphere. The crystalline structure of deposited and annealed films was deduced by SE and supported by X-ray diffraction (XRD). It was revealed that the anatase TiO_2_ film started to transform into rutile phase when the annealing temperature was up to 800 °C. In this paper, a constructive and effective method of monitoring the phase transition in ultrathin films by SE has been proposed when the phase transition is not so obvious analyzed by XRD.

## Background

Titanium dioxide (TiO_2_) became a promising material in different applications for its excellent optical and electrical properties and chemical stability such as large band gap, high refractive index, high dielectric constant, and highly active surface [1–4]. Traditionally, TiO_2_ pigment has almost been applied to every kind of paint due to its high refractive index [5, 6] and used as photocatalyst in the process of sterilizing, deodorizing, antifouling, and so on, which can convert light energy into electrical energy and chemical energy [6–8]. Moreover, the high dielectric constant (*k* ~ 80) of TiO_2_ allows it to take the place of traditional silicon dioxide (SiO_2_), used as capacitors in dynamic random access memory (DRAM) devices or ultrathin gate dielectric layers in field-effect transistors (FET) [9, 10]. Now, ultrathin TiO_2_ layers as a barrier with a mean thickness of <3 nm could enhance photovoltaic performance of the inverted organic solar cell, especially short-circuit current (*J*_sc_) and power conversion efficiency (PCE) [11]. Furthermore, metalenses at visible wavelengths with efficiencies as high as 86% have been demonstrated recently by using TiO_2_ materials, in which TiO_2_ could overcome the challenge of the high intrinsic losses in the visible range and realize the highly efficient metasurfaces in this region [12]. TiO_2_ has been one of the most studied materials in the last decades, and numerous research concerning TiO_2_ bulks and films have been already reported [1–4]. However, the research on the optical properties of TiO_2_ ultrathin films, especially below 20 nm, is still rare. Thus, detailed studies on the optical properties of sub-20-nm TiO_2_ films have become very important to the miniaturization of integrated photonic devices, the performance of solar cell devices, and even the application of metalenses [11–13].

TiO_2_ thin films can be prepared by various methods such as electron beam evaporation, laser-assisted evaporation, chemical vapor deposition, sol–gel process, sputtering, and atomic layer deposition (ALD) [14]. Among them, the ALD method has been usually adopted to grow ultrathin and high-*k* dielectrics owing to its advantages over other methods in completely precise thickness control, low processing temperature, low impurity content, no line-of-sight depositions, conformal coating capability on complex-shaped structures, and excellent thickness uniformity over large deposition area [1, 3, 15–17].

This work is focused on the study of optical properties of TiO_2_ ultrathin film, whose thickness is less than 20 nm. To achieve high-quality TiO_2_ ultrathin films, the ALD method has been employed to deposit samples on single crystal Si substrates. Furthermore, we investigate those films mainly by spectroscopic ellipsometry (SE), a powerful non-destructive and sensitive technique. The optical constants and optical band gap of the TiO_2_ ultrathin film are obtained, and the influence of nano size and temperature on the optical properties is also revealed. These results will be helpful to the applications of ultrathin TiO_2_ in optoelectronic semiconductor devices such as memory, field-effect transistors, and inverted organic solar cells. What is more, a novel method to monitor the phase transition in ultrathin films is proposed by deducing from the band gap evolution.

## Methods

TiO_2_ ultrathin films were fabricated on Si (100) substrates by ALD (Picosun R-series, Espoo, Finland). The substrates were heated using a resistive heating plate, providing temperatures up to 300 °C. Titanium tetraisopropoxide (TTIP) was the precursor for titanium, and H_2_O was the precursor for oxygen. The bubbler containing TTIP was heated to 80 °C. High purity N_2_ gas was used to purge the reactor chamber after each precursor pulse. Each ALD cycle consists of four steps: first, 0.5 s to pulse TTIP; then, 10 s to purge the chamber; next, 0.1 s to pulse H_2_O as the second reactant; eventually, 10 s to evacuate H_2_O [18].

After deposition, the as-deposited films were annealed by a rapid thermal process (RTP; AS-ONE, Montpellier, France) system at the temperatures of 400–900 °C with an increment of 100 °C in N_2_ atmosphere. Then, spectroscopic ellipsometry (SE; J. A. Woollam Co. M2000X-FB-300XTF, Lincoln, NE, USA) measurements were performed in order to examine the influence of microstructure characteristics on the optical properties of the films. All the films were characterized before and after annealing in the wavelength range from 200 to 1000 nm at the incident angle of 65 °C. The surface morphologies of films were studied using atomic force microscopy (AFM; Bruker Dimension Icon VT-1000, Santa Barbara, CA, USA) in tapping mode. The crystalline structures of films were also examined by X-ray diffraction (XRD; Bruker-AXS D8) and transmission electron microscope (TEM; FEI Tecnai G2 F20, Hillsboro, OR, USA). Besides, the reflection spectra (Lamda 950; PerkinElmer Instruments Co.) were measured to verify the band gap evolution and support the SE results.

## Results and Discussion

The TiO_2_ ultrathin films with different thicknesses were obtained by controlling the numbers of ALD cycles from 50 to 600 cycles. In order to identify the quality of as-deposited films and make SE measurements more effectively in characterizing the samples, information concerning the structure and morphology is an essential prerequisite. This information is conductive to establish the optical model and adopt a suitable dispersion law for calculating dielectric function in SE process [19, 20]. In consequence, the AFM, XRD, and TEM results of the as-deposited films were analyzed firstly.

**Fig. 1:**
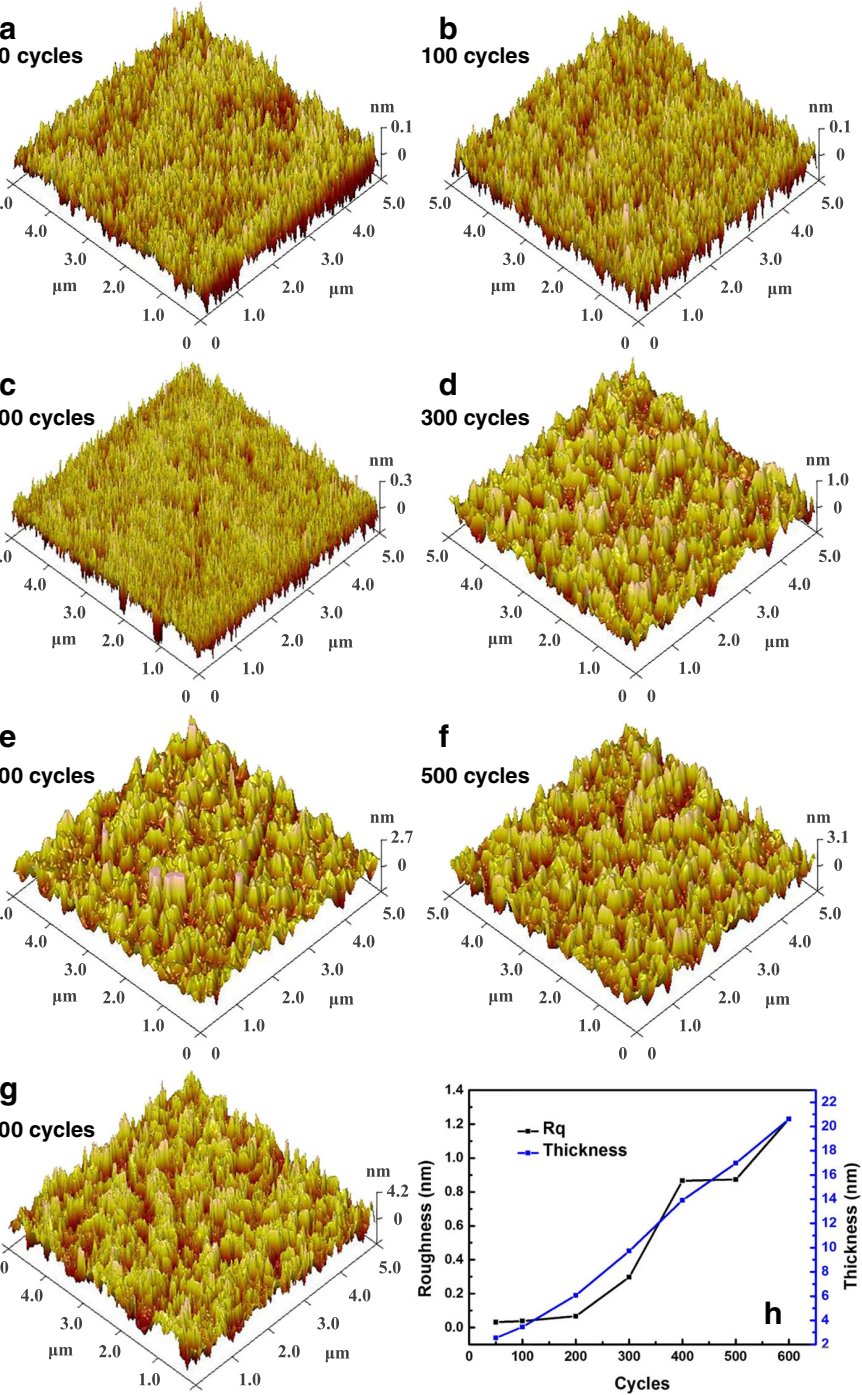
AFM micrographs of as-deposited TiO_2_ ultrathin films with different thicknesses. **a** 50 cycles. **b** 100 cycles. **c** 200 cycles. **d** 300 cycles. **e** 400 cycles. **f** 500 cycles. **g** 600 cycles. **h** Trend chart of roughness and thickness for the seven samples

The 3D AFM micrographs (scanning area 5 μm × 5 μm) are shown in Fig. 1. The surface morphologies of all films are smooth with different root mean square (RMS) roughness values (vary from 0.03 to 1.23 nm), which indicates that TiO_2_ ultrathin films were well fabricated (Fig. 1h). The small surface roughness can reduce the light scattering caused by rough surface in SE measurements [3].

**Fig. 2:**
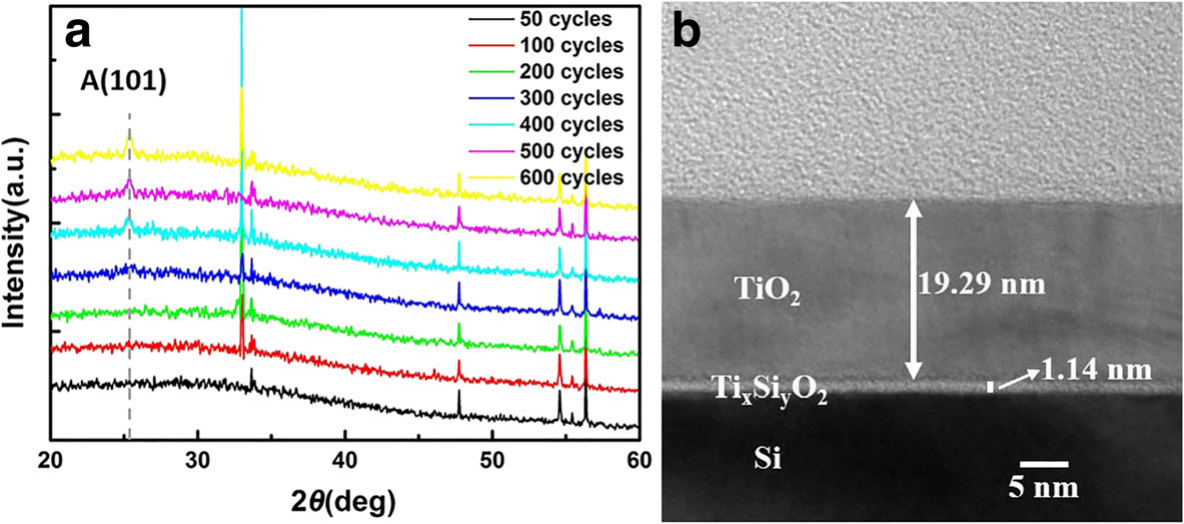
**a** XRD patterns for all films of different cycles grown on Si (100) substrates. **b** TEM micrograph for TiO_2_ ultrathin film of 600 cycles

The XRD patterns of all films with different thicknesses are given in Fig. 2a. The films, deposited below 300 ALD cycles, do not show any XRD peaks of TiO_2_, indicating those films have low crystallinity or even are amorphous in nature because TiO_2_ nanostructures in early ALD process evolve from amorphous layers to amorphous particles to metastable crystallites and finally to stable crystalline forms, which is a manifestation of the Ostwald–Lussac law [16]. When the ALD cycles are above 400, TiO_2_ ultrathin films begin to exhibit an anatase (101) peak at 2*θ* = 25.4° [21]. The intensity of anatase (101) peak is found to increase with increasing cycles, showing that the crystalline becomes better as film thickness increases. The mean grain size of the prepared TiO_2_ nano crystalline is 13.9 nm for the sample of 400 cycles, 17.1 nm for the sample of 500 cycles, and 20.5 nm for the sample of 600 cycles, calculated from the (101) diffraction peaks using Scherrer’s formula [22]. The gains with smaller size in thinner films could contribute to the close-packed nanocrystallites and larger crystalline volume fraction, resulting in lower *R*_q_. Hence, the increasing grain size can also explain the increasing surface RMS roughness varying with the increasing thickness in Fig. 1h [3]. Furthermore, on account of the phase transformation from amorphous state to crystalline state as implied in Fig. 2a, an increased RMS roughness of ~1 nm is observed in Fig. 1 when the cycles are above 400. The TEM result of 600 cycles TiO_2_ is provided in Fig. 2b. There is no denying the fact that an interface layer exists between Si and TiO_2_ obviously and the thickness of interface layer is about 1 nm.

Then, the optical properties of TiO_2_ ultrathin films were investigated by spectroscopic ellipsometry (SE). SE is well-known for its non-contact and non-destructive measurement and applies in extracting the thickness, optical constants, and band gap by a proper optical model [23]. The ellipsometric parameters *Ψ* and Δ are defined by the ellipsometric ratio *ρ* as [23, 24]:

1 $$ \rho ={r}_s/{r}_p= \tan \varPsi \exp \left( j\Delta \right) $$

where *r*_*p*_ and *r*_*s*_ are the complex reflection coefficients of polarized light parallel and perpendicular to the incidence plane, respectively. As the surface roughnesses of TiO_2_ films are very small according to the AFM micrographs and the cross-sectional structure shown in TEM micrographs, an optical model comprising air/TiO_2_/Ti_x_Si_y_O_2_/Si is established in the fitting process to reduce the uncertainty of fitting [25].

The Forouhi–Bloomer (F-B) dispersion model is considered to describe the optical constants of TiO_2_ [23]. The F-B dispersion model is of benefit to determining the refractive index *n* and extinction coefficient *k* values accurately, which could provide a decent description of the excitations near the absorption threshold in disordered dielectrics. F-B dispersion model is described as follows [23, 26]:

2 $$ n(E)= n\left(\infty \right)+{\displaystyle \sum_i}\frac{{B_0}_i E+{C_0}_i}{E^2-{B}_i E+{C}_i} $$

3 $$ k(E)={\displaystyle \sum_i}\frac{A_i\left( E-{E}_g\right)}{E^2-{B}_i E+{C}_i} $$

where *n*(∞) is the refractive index when photon energy *E* → ∞, *E*_*g*_ is the band gap of TiO_2_, ${B_0}_i=\frac{A_i}{Q_i}\left[-\frac{{B_i}^2}{2}+{E}_g{B}_i-{E_g}^2+{C}_i\right]$, ${C_0}_i=\frac{A_i}{Q_i}\left[\left({E_g}^2+{C}_i\right)\frac{B_i}{2}-2{E}_g C\right]$, ${Q}_i=\frac{1}{2}{\left(4{C}_i-{B_i}^2\right)}^{1/2}$, and *A*_*i*_, *B*_*i*_, *C*_*i*_ are positive nonzero parameters characteristic of the medium such that 4*C*_*i*_ − *B*_*i*_^2^ > 0. In addition, to characterize the fitting precision, the root mean square error (RMSE) is described as [23, 24]:

4 $$ \mathrm{RMSE} = \sqrt{\frac{1}{2 N- M-1}{\displaystyle \sum_{i=1}^N\left[{\left({\varPsi}_i^{\mathrm{cal}}-{\varPsi}_i^{\exp}\right)}^2+{\left({\varDelta}_i^{\mathrm{cal}}-{\varDelta}_i^{\exp}\right)}^2\right]}} $$

where *N* is the number of data points in the spectra, *M* is the number of variable parameters in the model, and “exp” and “cal” represent the experimental and the calculated data, respectively [27]. The fitting results are considered ideal until the RMSE value is less than 1 [23].

The thicknesses of films calculated by SE are listed in Table 1. As we can see, all the samples except for those of 50 cycles grew at a stable speed of ~0.34 Å/cycle. In order to study the cause of different growth rates of those of 50 cycles, we also have studied the growth rate of films with ALD cycles below 50. It is found that the growth rate is still changeable and varies from 0.36 to 0.44 Å/cycle. Therefore, we hold the opinion that two reasons could cause that. On the one hand, the early ALD growth process is not stable and is in the process of nucleation. On the other, SE results exist at a certain degree of calculation error for ~0.3 nm, which could lead to a considerable calculation error to growth rate (~0.1 Å/cycle when ALD cycles are below 50).

**Table 1:** Thicknesses of TiO_2_ ultrathin films calculated by SE

ALD cycles	50	100	200	300	400	500	600
Thickness (nm)	2.55	3.45	6.05	9.72	13.90	16.97	20.61

**Fig. 3:**
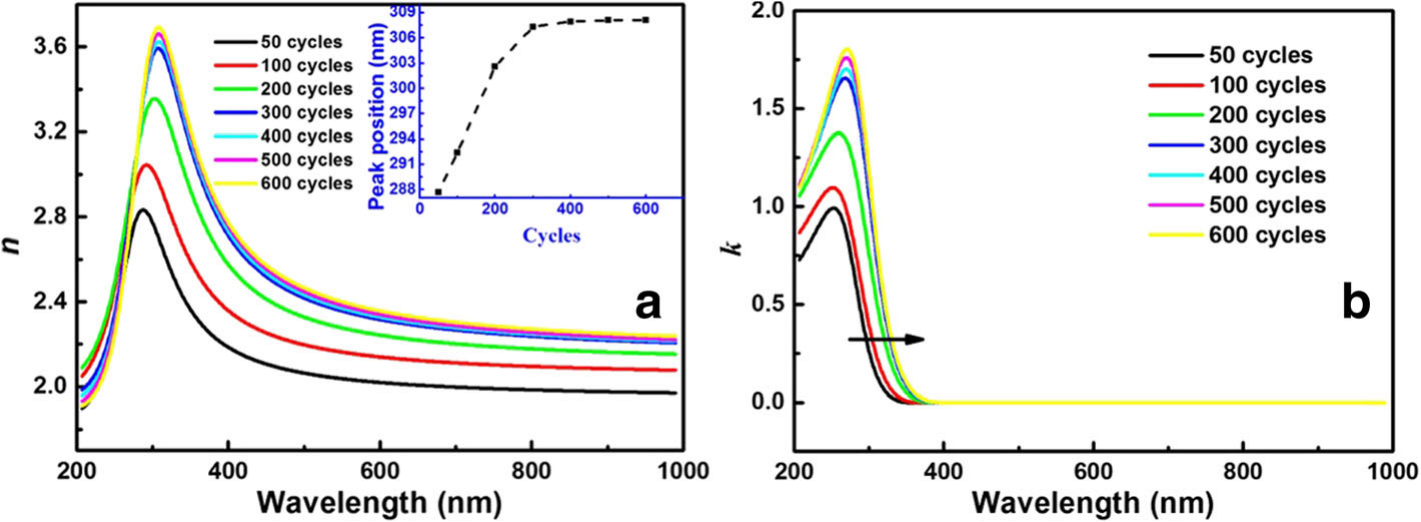
**a** Refractive index *n* and **b** extinction coefficient *k* spectra for TiO_2_ ultrathin films with different thicknesses. The *inset* of **a** shows the plot of peak position versus ALD cycles

Figure 3 displays the dispersion curves of refractive indices (*n*) and extinction coefficients (*k*). It is noticed in Fig. 3a that refractive indices increase with the increasing thicknesses of films, which is most caused by the density of films [28]. The volume ratio of air in the layer decreases when the thickness of film increases [13]. Moreover, the peak of refractive index occurs redshift with the increase of thickness, which is due to the redshift of the anomalous dispersion region (inset of Fig. 3a). Figure 3b explains that extinction coefficients increase gradually with the increasing thickness, consistent with the tendency of refractive indices. The absorption edge also shows an obvious redshift, indicating a decrease of band gap.

**Fig. 4:**
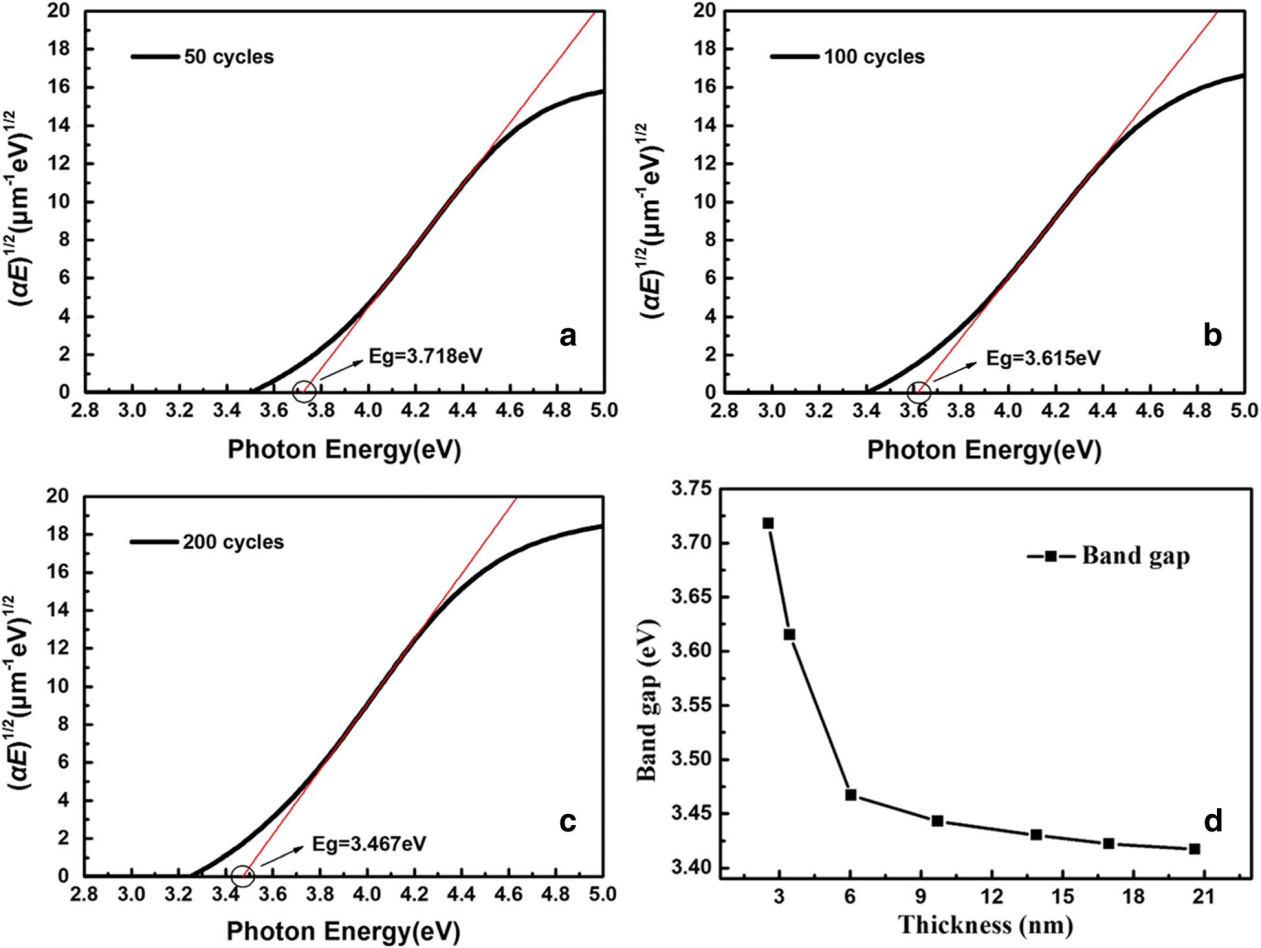
Plots of (*αE*)^1/2^ vs. *E* for **a** 50 cycles, **b** 100 cycles, and **c** 200 cycles. **d** Trend chart of band gap for seven samples

In order to quantify the variation of band gap, linear extrapolation of absorption coefficient *α* was conducted to determine the band gap energy evolution of TiO_2_ films with the formula described as [23]:

5 $$ \alpha =\frac{4\pi k}{\lambda}=\frac{K{\left( E-{E}_{\mathrm{g}}\right)}^m}{E} $$

where *k* is the extinction coefficient, *λ* is the incident wavelength, *K* is the constant, *E* is the photon energy, *E*_g_ is the band gap, and *m* is the number decided by the transition process, specifically divided into two kinds of situations: *m* equals to 1/2 for direct transition and 2 for indirect transition [23, 29]. The amorphous and anatase TiO_2_ have an indirect band gap, so the *m* should be 2. Figure 4 shows the plots of (*αE*)^1/2^ vs. *E* for three samples, and Table 2 lists all the band gaps of seven samples. It is obviously found that the value of band gap declines from 3.718 to 3.417 eV with the increasing thickness (Fig. 4d), as a result of quantum confinement effect [13, 30]. The decrease of the size of material leads to the increase of the band gap. Meanwhile, the smaller band gap in thicker film could cause a redshift of the anomalous dispersion region as illustrated in Fig. 3.

**Table 2:** Band gaps of TiO_2_ ultrathin films

Cycles	50	100	200	300	400	500	600
Band gap (eV)	3.718	3.615	3.467	3.443	3.430	3.422	3.417

**Fig. 5:**
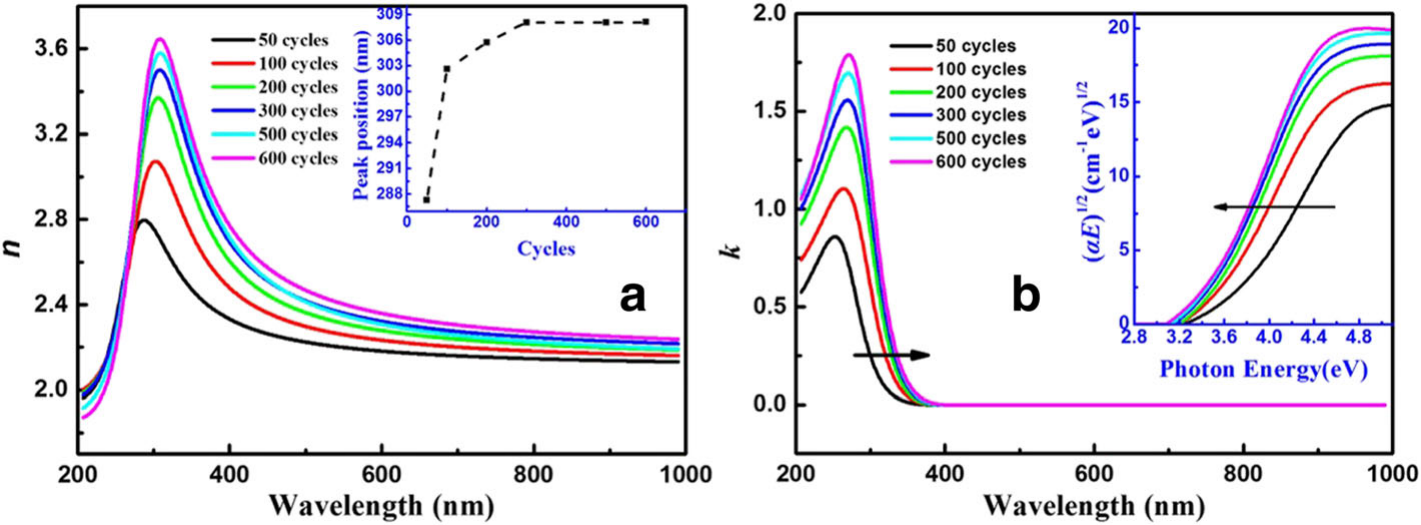
**a** Refractive index *n* spectra and **b** extinction coefficient *k* spectra for TiO_2_ ultrathin films with different thicknesses after annealing at 400 °C. The insert of **a** shows plot of peak position versus ALD cycles. The insert of **b** shows plots of (*αE*)^1/2^ vs. *E* for TiO_2_ ultrathin films with different thicknesses after annealing at 400 °C

To investigate the temperature dependence of optical properties of TiO_2_ ultrathin films, the samples were annealed at the temperatures of 400, 500, 600, 700, 800, and 900 °C, respectively. Figure 5a, b shows the optical constants of TiO_2_ ultrathin films with different thicknesses after annealing at the same temperature of 400 °C. It can be found that the rapid thermal process (RTP) does not destroy the regularity of optical constants varying with thickness. The refractive index and the extinction coefficient become higher with the increase of the thickness, while the band gap of TiO_2_ ultrathin films appears to have an opposite trend, as inferred from the inset of Fig. 5b.

**Fig. 6:**
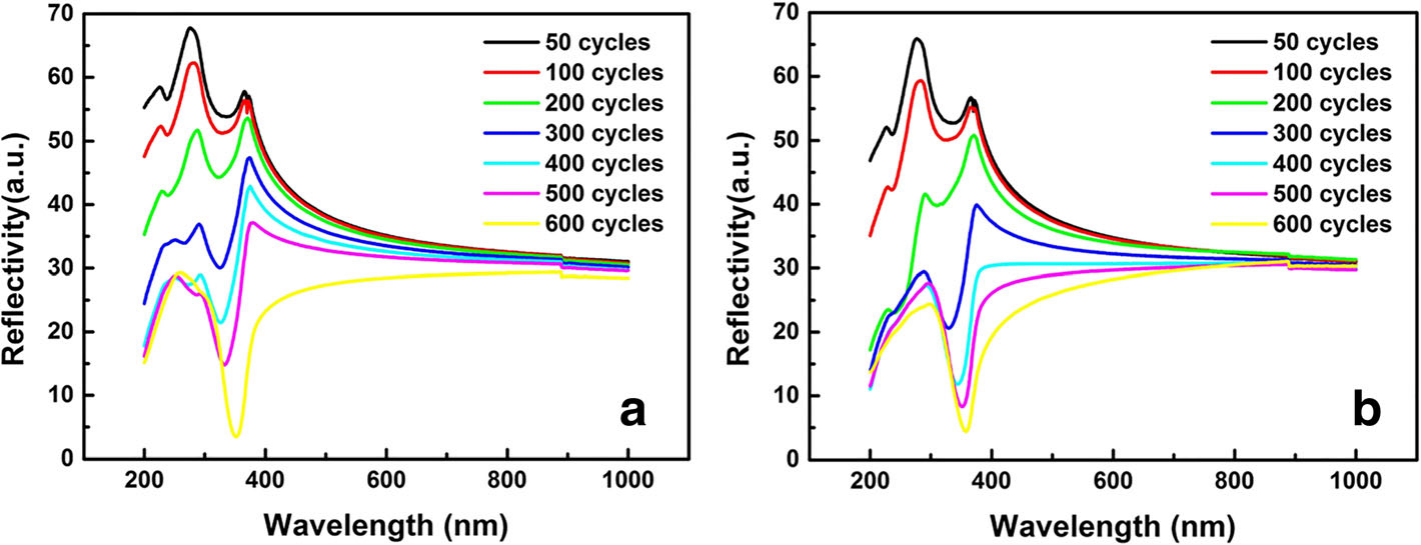
Reflection spectra of TiO_2_ ultrathin films **a** before and **b** after annealing at 900 °C

Figure 6 displays the reflection spectra of TiO_2_ ultrathin films before and after annealing at 900 °C. It can be seen clearly that the absorption edge sinks after annealing, especially for samples of 300, 400, and 500 cycles, indicating that the annealing process is very effective [31]. Furthermore, absorption edge has a redshift with increasing thickness before and after annealing, which illustrates the decrease of band gap. This also verifies the regularity of band gap evolution changing with thickness obtained via SE before.

**Fig. 7:**
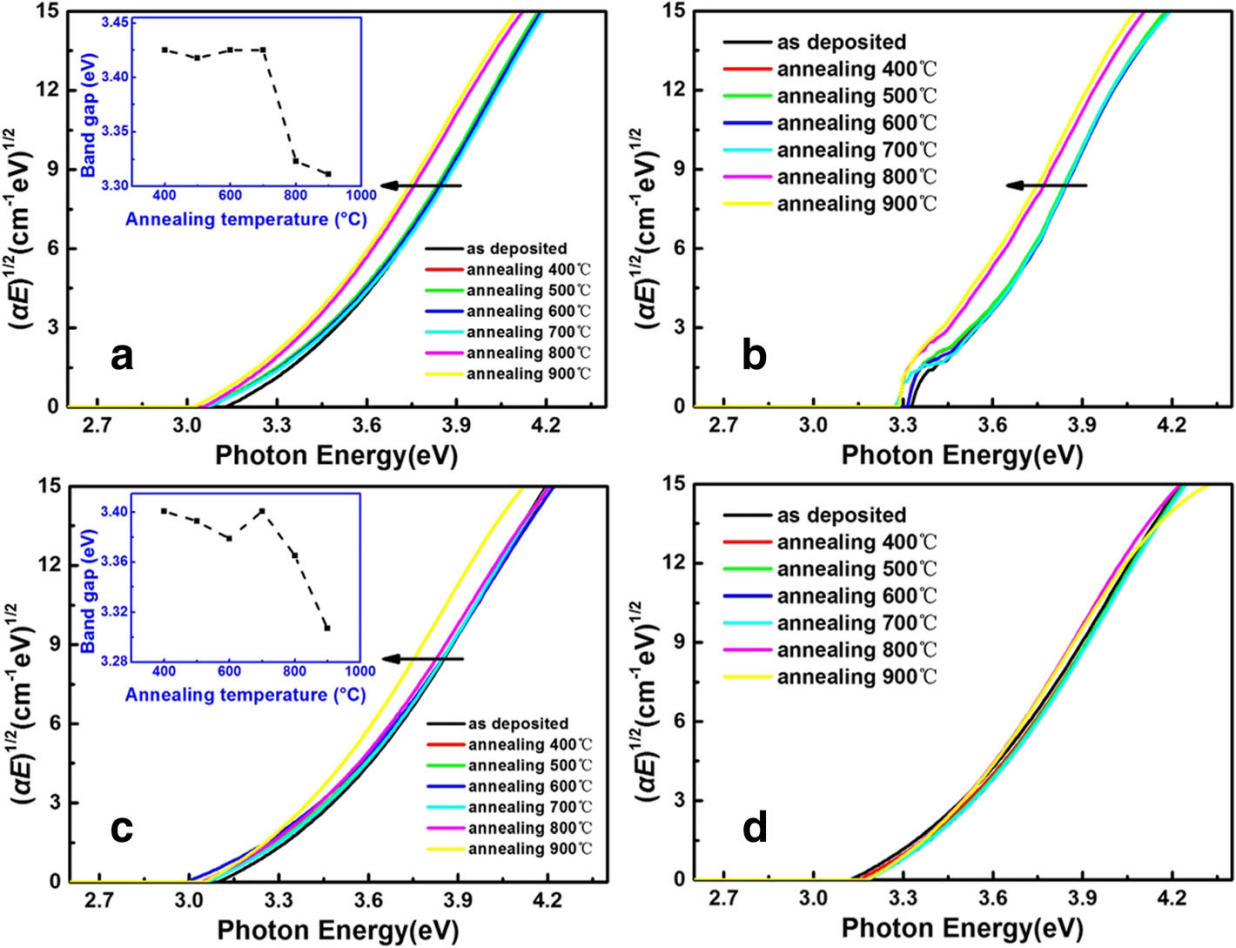
Plots of (*αE*)^1/2^ vs. *E* for TiO_2_ ultrathin films of **a** 600 cycles caculated by the F-B dispersion model, **b** 600 cycles caculated by the point-by-point data inversion method, **c** 500 cycles caculated by the F-B dispersion model, and **d** 300 cycles caculated by the F-B dispersion model. The *inset* of **a** shows the trend chart of band gap for 600 cycles, and the *inset* of **c** shows the trend chart of band gap for 500 cycles

With the purpose of studying the variation of optical properties under different annealing temperatures, samples of 600 cycles were selected and annealed in the range of 400 to 900 °C with an increment of 100 °C. As can be seen in Fig. 7a, absorption edge suddenly presents a shift and the band gap of TiO_2_ ultrathin films decreases suddenly (inset of Fig. 7a) when the films were annealed at 800 and 900 °C. Then, the point-by-point data inversion method is employed to verify the accuracy of the reduction of band gap, as shown in Fig. 7b. It is found that the annealing temperature of 800 and 900 °C do cause a decrease of band gap.

Meanwhile, samples of 500 and 300 cycles after annealing were also selected to conduct SE analysis, as shown in Fig. 7c, d. The TiO_2_ film of 500 cycles also appears to have a sharp decrease of band gap at 900 °C (inset of Fig. 7c), while the film of 300 cycles does not. It is conjectured that crystalline phase has been changed from anatase to rutile phase by high temperature annealing for the samples of 600 and 500 cycles, while the samples of 300 cycles are still amorphous [4, 32]. According to the previously reported research, the band gap of anatase bulk TiO_2_ is 3.23 eV, while that of the rutile bulk TiO_2_ is 3.02 eV [5], which is in accordance with the decrease of band gap described above, whereas there is no such phenomenon in the TiO_2_ ultrathin film with ALD cycles fewer than 300, owing to no crystallization in extremely thin films.

**Fig. 8:**
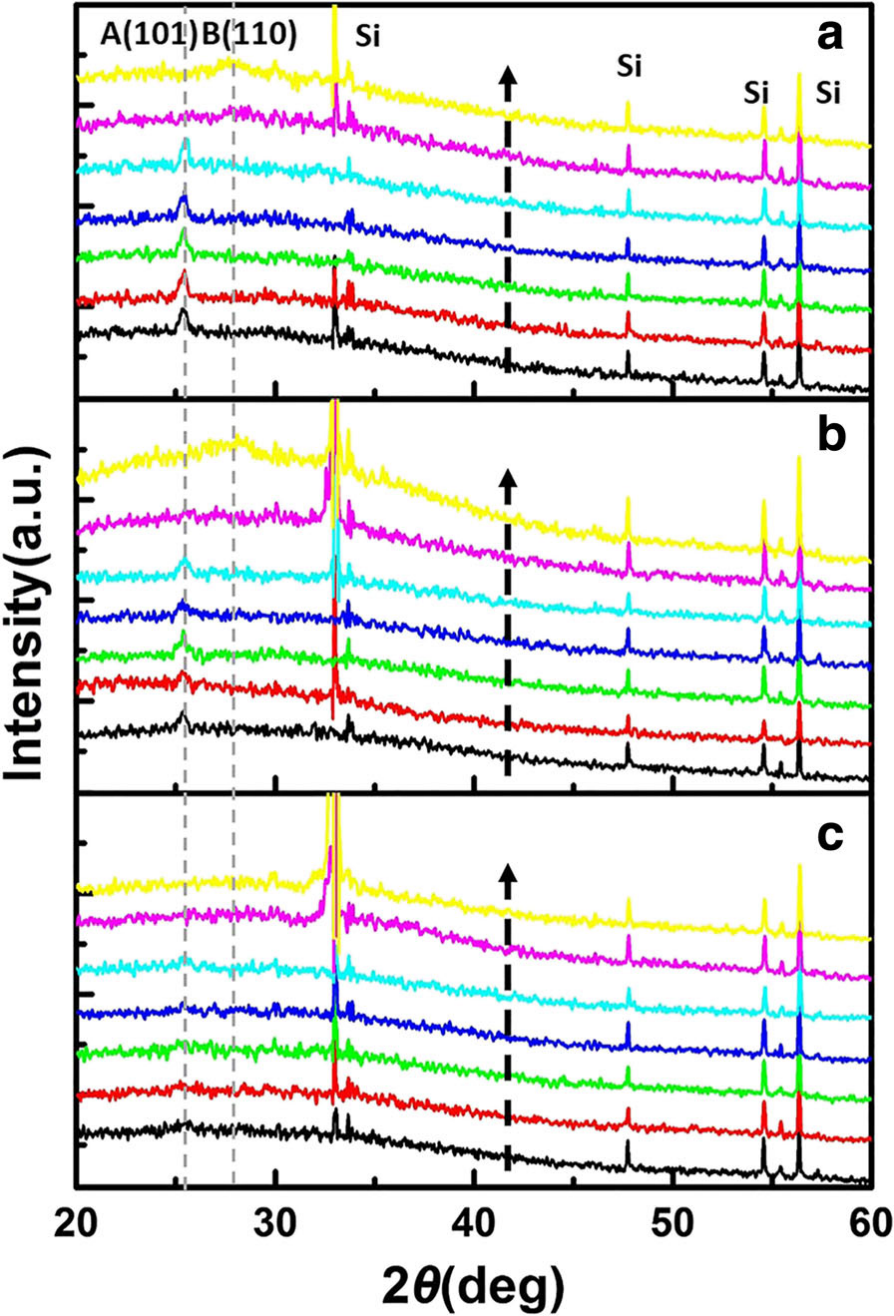
XRD patterns for films of **a** 600, **b** 500, and **c** 300 cycles before and after annealing. The annealing temperatures are increasing from *bottom* to *top*: as deposited (*black*), 400 °C (*red*), 500 °C (*green*), 600 °C (*blue*), 700 °C (*cyan*), 800 °C (*magenta*), and 900 °C (*yellow*)

Figure 8a presents the XRD patterns for films of 600 cycles after annealing, in which exists an obvious anatase (101) peak at the annealing temperature of 400–700 °C. When the annealing temperature is as high as 800 °C, the anatase peak disappears suddenly and a rutile (110) peak emerges gradually at 2*θ* = 27.5° [4]. It has been proved that the shift of band gap in Fig. 7a, b is caused by phase transformation.

The XRD patterns for films of 500 and 300 cycles after annealing are also given in Fig. 8. For the former films, the anatase (101) peak disappears when the annealing temperature is up to 800 °C and the rutile (110) peak appears instead at 900 °C (Fig. 8b). And the latter films, as can be seen in Fig. 8c, are always amorphous before and after annealing. Thus, these results are consistent with the calculated results of SE.

## Conclusions

In summary, TiO_2_ ultrathin films with high purity were grown in a self-limited ALD growth mode by using TTIP as Ti precursors on silicon substrates at a substrate temperature of 300 °C. The TiO_2_ layers were characterized with respect to microstructure, composition, optical constants, and optical band gap by atomic force microscopy, X-ray diffraction, reflectance spectroscopy, and spectroscopic ellipsometry.

The effect of thickness on the optical properties of TiO_2_ ultrathin films has been investigated. The refractive index and absorption coefficient increase with the increasing film thickness, attributed to the effect of pore. On the other hand, the quantum size effect could cause the variation of band gap. Then, the as-deposited films were annealed at the temperature of 400 to 900 °C in N_2_ atmosphere. It is found that thermal annealing does not destroy the regularity of optical constants. As for the TiO_2_ ultrathin films with ALD cycles above 400, high temperature around 800 °C transforms the anatase TiO_2_ to rutile TiO_2_ via deducing from band gap evolution, which provides a novel method to detect the phase transition in ultrathin films. The results in this paper about TiO_2_ ultrathin films will play a role in the future application of optoelectronic devices.

## Abbreviations

AFM: Atomic force microscopy
ALD: Atomic layer deposition
DRAM: Dynamic random access memory
F-B: Forouhi–Bloomer
FET: Field-effect transistors
PCE: Power conversion efficiency
RMS: Root mean square
RMSE: Root mean square error
RTP: Rapid thermal process
SE: Spectroscopic ellipsometry
SiO_2_: Silicon dioxide
TEM: Transmission electron microscope
TiO_2_: Titanium dioxide
TTIP: Titanium tetraisopropoxide
XRD: X-ray diffraction
